# Antigenic Divergence from the Seasonal Vaccine of the Influenza Virus Strains Circulating in Romania During Three Successive Seasons (2021–2024)

**DOI:** 10.3390/microorganisms12112363

**Published:** 2024-11-19

**Authors:** Ovidiu Vlaicu, Leontina Banica, Robert Hohan, Marius Surleac, Dragoş Florea, Victor Daniel Miron, Andreea Tudor, Oana Săndulescu, Anca Cristina Drăgănescu, Dan Oțelea, Simona Paraschiv

**Affiliations:** 1 National Institute for Infectious Diseases “Prof. Dr. Matei Bals”, 021105 Bucharest, Romania; vlaicu.ovidiu@yahoo.com (O.V.); hohan.robert@gmail.com (R.H.); marius.surleac@gmail.com (M.S.); dragos.florea@umfcd.ro (D.F.); victor.miron@umfcd.ro (V.D.M.); tudor.andreea0711@gmail.com (A.T.); oana.sandulescu@umfcd.ro (O.S.); anca.draganescu@umfcd.ro (A.C.D.); dotelea@mateibals.ro (D.O.); 2 Carol Davila University of Medicine and Pharmacy, 050474 Bucharest, Romania; 3 Research Institute of the University of Bucharest, University of Bucharest, 050095 Bucharest, Romania

**Keywords:** influenza virus, vaccine efficacy, antigenic relatedness, hemagglutinin sequences, influenza clades, phylogenetic analysis, Romania

## Abstract

Influenza viruses continue to be an important public health threat. Vaccination is the most effective measure to control the influenza virus circulation. However, these viruses are continuously evolving through antigenic drift/shift, and thus the vaccine efficiency is affected. The aim of this study was to characterize the viral strains circulating in Romania, in a population with declining vaccination coverage, during the last three cold seasons by evaluating the hemagglutinin antigenic relatedness to the vaccine strains. All the available sequences collected between August 2021 and June 2024 were analyzed by using phylogenetic analysis and the P_epitope_ model to predict vaccine efficacy. The results showed that the 2021/2022 influenza season was dominated by the circulation of highly diverse clades of A(H3N2) viruses with high mutational divergence as compared to the vaccine strain, which might contribute to the reduction in vaccine efficacy. During the 2022/2023 influenza season, both influenza A and B viruses were reported, with few antigenic site mutations. The 2023/2024 influenza season was dominated by the circulation of influenza A viruses: A/H1N1pdm09 clade 6B.1A.5a.2a and A/H3N2 clade 2a.3a.1. The clade 2a.3a.1 also showed high variability when compared to the vaccine strain, presumably leading to reduced vaccine efficacy. This study illustrates the high diversity of influenza viruses circulating in a population with low vaccination coverage during the previous cold seasons. The viral diversity impacted vaccine efficacy, hence the need for public health programs to increase vaccine uptake and improve vaccine formulation in order to limit viral transmission.

## 1. Introduction

Influenza viruses circulate worldwide, with seasonality from April to September in the Southern Hemisphere and October to May in the Northern Hemisphere. Due to their widespread circulation, those viruses cause an important number of infections each season; according to the World Health Organization (WHO), around a billion influenza cases are registered each year, with three to five million of them severe [1]. The impact of an occasional outbreak with a new virus subtype due to antigenic shift (through the reassortment of the genetic material from different viral subtypes) is even higher. The human seasonal epidemics are caused by influenza A and B viruses that naturally acquire mutations in their hemagglutinin (HA) and neuraminidase (NA), leading to escape from neutralization through antibodies produced by previous immunizations [2]. The antigenic drift (progressive accumulation of point mutations) is the more common mechanism responsible for the high variability of influenza viruses which implies the constant need to update the vaccination formula [3].

The WHO makes yearly recommendations for the composition of the influenza vaccine by analyzing molecular and antigenic surveillance data. In recent years, the vaccine included two influenza A strains (H1N1 and H3N2) and two strains of influenza B (lineages Victoria and Yamagata) [4]. The selection of influenza strains is often challenging and involves several steps: the identification of the circulating viruses, the evaluation of the immune protection induced by previous vaccinations, and its ability to confer cross-protection [5]. Influenza A and B genomes are composed of eight separated negative single-stranded RNA segments coding for RNA polymerase (polymerase basic 2—PB2, polymerase basic 1—PB1, polymerase acidic—PA), non-structural proteins (NS), hemagglutinin (HA), neuraminidase (NA), matrix proteins (MP), and nucleoprotein (NP) [6]. HA is a glycoprotein located on the virus surface, which is responsible for receptor binding and fusion with the cell membrane [7]. It consists of two domains, the globular head domain (HA1), which includes the antigenic sites, and the stem domain (HA2). Five antigenic sites were described for influenza A viruses, namely Sa, Sb, Ca1, Ca2, and Cb for A(H1N1)pdm09 [8] and A through E for A(H3N2) [9], and four major HA antigenic sites were proposed for influenza B viruses (120-loop, 150-loop, 160-loop, and 190-helix) [10].

Since 2018, The National Institute for Infectious Diseases (NIID) “Prof. Dr. Matei Balș” is one important national site where the molecular surveillance of influenza viruses is constantly performed by whole-genome sequencing as part of the Global Influenza Hospital Surveillance Network (GIHSN) [11]. Romania reported low rates of influenza vaccination, with an overall coverage of 5.7% during the last season and only 16.4% in the elderly population (≥65 years old), which is far from the WHO recommendation of 75% in this particular risk group [12,13]. No data are available on vaccine uptake in children; however, free vaccine doses are also covered through the national vaccination program targeting this risk group. Moreover, the results of vaccine effectiveness reported for the 2021/2022 flu season by the Development of Robust and Innovative Vaccine Effectiveness (DRIVE) study group, which included our center, showed the lowest vaccine effectiveness in children group (6 months–6 years) [14].

Vaccine-induced antibodies to HA are considered the major effectors of protection and therefore the analysis of the variability in this protein is often used as a surrogate for estimating vaccine efficacy. The purpose of this study was to assess the diversity in the HA influenza gene of strains circulating in Romania in recent years (2021–2024) and the antigenic relatedness with the vaccine strains in order to estimate the efficacy of the seasonal vaccine by using a previously validated mathematical model (P_epitope_) [9].

## 2. Materials and Methods

### 2.1. Clinical Sample Selection

Clinical samples were obtained from patients (children and adults) hospitalized at NIID “Prof. Dr. Matei Balș”, who presented with influenza-like illness (ILI) or severe acute respiratory infection (SARI) symptoms during three influenza seasons (2021–2024). The criteria for inclusion of patients in the study were established according to the adapted GIHSN protocol, described in detail previously [15,16]. A total of 2544 patients were evaluated. The influenza positivity rate was 42.9%. Children accounted for 60.4% of positive cases, while 9.5% were elderly persons. The overall influenza vaccination rate was 4.9% among those who tested positive.

The study was approved by the Bioethics Committee of the National Institute for Infectious Diseases “Prof. Dr. Matei Balș”, Bucharest, Romania. This study was conducted in accordance with the Declaration of Helsinki. All patients in the study provided written informed consent.

### 2.2. Whole-Genome Sequencing and Bioinformatics Analysis

A total number of 367 respiratory swabs collected on viral transport media (VTM) (COPAN ITALIA S.P.A., Brescia, Italy) were used for viral RNA extraction using QiAmp DSP Virus (Qiagen, Hilden, Germany). Reverse transcription and PCR amplification were performed using the primers previously described for influenza A viruses and influenza B viruses [17,18]. The generated amplicons covering the whole genome of influenza viruses were further processed with an Illumina DNA Prep Kit (Illumina, San Diego, CA, USA) according to the manufacturer’s recommendations. Sequencing was performed on the MiSeq platform (Illumina) by using the paired-end shotgun strategy. The reads were assembled by a double approach protocol (de novo and reference mapping) as previously described [19]. A reference sequence for each viral type/subtype was used for mapping purposes. In total, 367 respiratory samples were successfully sequenced through this method during the study period (August 2021–June 2024) and uploaded to the GISAID database.

### 2.3. Hemagglutinin Sequence Datasets and Phylogenetic Analysis

In order to build a representative dataset for Romania, we included all influenza HA sequences that were deposited in the GISAID database and reported in Romania during the study period. After removing duplicates and deleting low-quality sequences (with gaps or incomplete), the datasets consisted of 444 influenza A(H1N1)pdm09 sequences; 450 A(H3N2) sequences; and 101 influenza B, lineage Victoria. These sequences were mainly reported by two national sites, the Molecular Diagnostics Laboratory from the NIID “Prof. Dr. Matei Bals” (367 sequences generated as described above) and the National Institute of Research and Development for Microbiology and Immunology ‘Cantacuzino’ (*n* = 628). The sequences belonging to each subtype/lineage were aligned with reference sequences, namely clade-defining sequences indicated by the Nextstrain platform [20], sequences of the vaccine strains recommended for the 2021/2024 influenza seasons, and other similar sequences to the Romanian ones that were retrieved, by performing a BLAST analysis. The final alignments were used to perform phylogenetic analysis by using the maximum likelihood method implemented in FastTree [21], with generalized time-reversible (GTR) models of nucleotide evolution. The trees were visualized and annotated with FigTree v1.4.4 [22].

### 2.4. Antigenic Relatedness to Vaccine Strains

A mathematical model, P_epitope_, that incorporates multiple parameters characterizing human antibody recognition of HA antigens was previously developed and validated [9,23]. The P_epitope_ model [9] was used to compute the relatedness of the circulating strain with the seasonal vaccine strains recommended by the WHO for the 2021–2024 influenza seasons. The following vaccine strains were used as a reference for the amino acid mutation analysis: A/Victoria/2570/2019, A/Victoria/4897/2022 (for A(H1N1)pdm09); A/Cambodia/e0826360/2020, A/Darwin/9/2021 (for A/H3N2); and B/Washington/02/2019, B/Austria/1359417/2021 (for influenza B lineage Victoria). The B-cell antigenic epitopes were identified for each subtype based on previous reports. Thus, for influenza A/H1N1pdm09, five antigenic epitopes were considered: Sa (amino acid position excluding signal peptide 124, 125, 153–157, 159–164), Sb (183–194), Ca1 (166–170, 203–205, 235–237), Ca2 (137–142, 144–147, 221–222), and Cb (70–75) [8]. The amino acid positions of the five antigenic epitopes (antigenic site A, site B, site C, site D, and site E) of influenza A(H3N2) taken into account for the analysis were those previously described [9]. The B-cell epitopes for influenza B/lineage Victoria included the 120-loop (116–137 amino acids), 150-loop (141–150 amino acids), 160-loop (position 160–172) and 190-helix (position 193–202) [10]. The mutations in these sites were used to identify the dominant epitope and to estimate vaccine efficacy (VE) based on the P_epitope_ model. This model assumes an efficiency of 53% for the A(H1N1)pdm09 component of the seasonal vaccine, with an estimated efficacy given by the formula E = −1.19 × P_epitope_ + 0.53, where P_epitope_ is the ratio between the number of mutations to the number of amino acids in the epitope. The dominant epitope is considered the one with the highest ratio. For influenza A/H3N2, the mathematical formula to estimate the antigenic relatedness between sequences is E =  −2.47 × P_epitope_ + 0.47 and assumes an efficacy of 47% when no mutations are found, while for influenza B, the efficacy is given by the formula E = −0.864 × P_epitope_ + 0.6824, with an estimated efficacy of 68.24% for the seasonal vaccine for this component [24].

## 3. Results

### 3.1. A(H1N1)pdm09 Variability and Phylogenetic Relationship of Circulating Strains in Romania (2021–2024)

**Figure 1:**
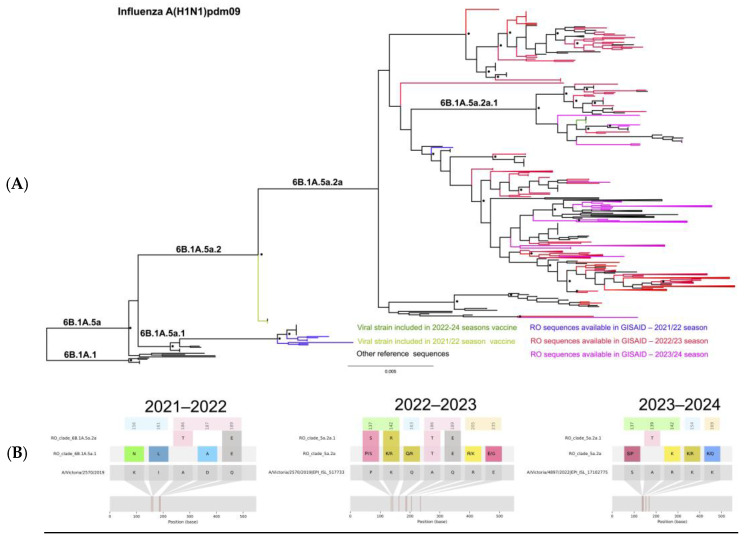
(**A**) Phylogenetic relatedness between A(H1N1)pdm09 sequences reported in Romania between 2021 and 2024. Shimodaira Hasegawa (SH) support values higher than 0.9 are indicated at nodes with an asterisk *. Romanian (RO) sequences are depicted according to each season: in blue (2021/2022), red (2022/2023), and magenta (2023/2024). (**B**) HA amino acid variability across clades circulating during the studied seasons. The antigenic epitope positions are highlighted in light blue (Sa), pink (Sb), light orange (Ca1), and light green (Ca2).

The A(H1N1)pdm09 phylogenetic tree shows that several different viral variants were circulating simultaneously in the two cold seasons studied. The 2021/2022 season was different, presenting only one main viral variant (marked in dark blue). The results of phylogenetic analyses performed on the HA sequences of the influenza A(H1N1)pdm09 strains circulating in Romania during the study period are presented in Figure 1A.

The 2021/2022 influenza season was characterized by a low prevalence of infections with influenza virus A(H1N1)pdm09. During this season, only nine sequences for A(H1N1)pdm09 from Romania (depicted with blue in Figure 1) were deposited in the GISAID database; most of these viruses (8/9) belonged to clade 6B.1A.5a.1 and clustered together with the 2020/2021 vaccine strain (A/Guangdong-Manon/SWL1536/2019), while one clustered with the Southern Hemisphere 2023 vaccine strain (A/Sydney/5/2021) in 6B.1A.5a.2a. Compared to the corresponding vaccine strain (A/Victoria/2570/2019, shown in light green), the variability assessed in the antigenic sites showed that the sequences belonging to clade 5a.1 presented two mutations in antigenic site Sa (K156N, I161L) and two other in Sb (D187A, Q189E). The impact of these mutations on VE was evaluated with the P_epitope_ model, and the result indicated a reduction in efficacy to 33.17%.

While analyzing the HA sequences of the viruses circulating in the 2022/2023 season in Romania (in red in Figure 1A), we observed that most of the A(H1N1)pdm09 strains belonged to clade 6B.1A.5a.2a and only few sequences (11/277) clustered in clade 6B.1A.5a.2a.1. All the sequences from this season, regardless of clade distribution, presented two mutations (A186T and Q189E) in the Sb antigenic site, the dominant epitope, with an estimated VE on these sequences reduced to 33.17%. Fifty-two of these sequences (18%) also presented one mutation (P137S) in the antigenic site Ca2.

The 2023/2024 influenza season (in magenta) was characterized by the predominance of clade 6B.1A.5a.2a, and only 5/158 sequences clustered in clade 6B.1A.5a.2a.1 together with the corresponding sequence of the 2023/2024 vaccine strain (A/Victoria/4897/2022 marked in dark green). Most of the viral sequences belonging to clade 5a.2a (91%) presented two mutations in the Ca2 antigenic site, namely S137P and R142K, while ten sequences presented only R142K. The estimated impact of these two mutations is a reduction in VE to 33.17%.

### 3.2. A(H3N2) Variability and Phylogenetic Relationship of Circulating Strains in Romania (2021–2024)

**Figure 2:**
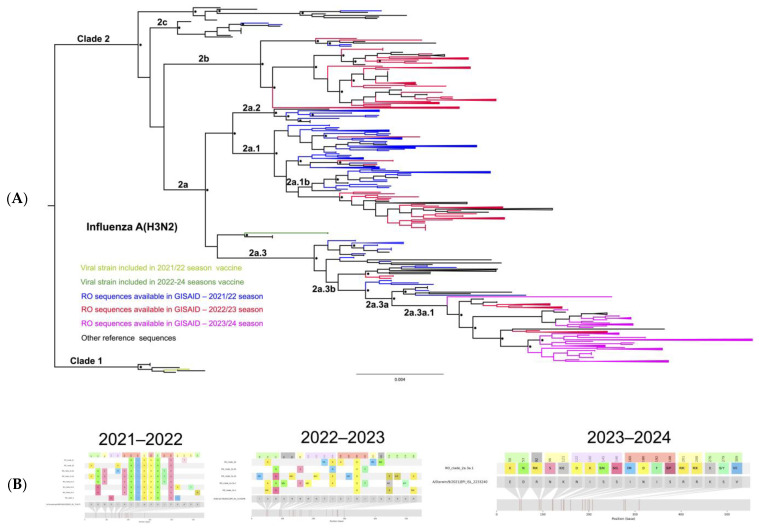
(**A**) Phylogenetic relatedness between A(H3N2) sequences reported in Romania in the study period. Shimodaira Hasegawa (SH) support values higher than 0.9 are indicated at nodes with an asterisk *. Romanian (RO) sequences are depicted according to each season: in blue (2021/2022), red (2022/2023), and magenta (2023/2024). (**B**) HA amino acid variability across clades circulating during the studied season. The antigenic epitope positions are highlighted in lavender (epitope A), red (epitope B), green (epitope C), yellow(epitope D), and gray (epitope E).

The sequences belonging to the viruses circulating during the 2021/2022 flu season in Romania are represented in blue in Figure 2A and are dispersed in multiple clades/subclades throughout the phylogenetic tree. According to the results presented in Figure 2A,B, the 2021/2022 influenza season in Romania was characterized by the circulation of highly diverse influenza A/H3N2 strains. The dominant clade was 2a.1 (104/186). However, all the strains circulating during the 2021/2022 influenza season were highly mutated as compared to the vaccine sequence (A/Cambodia/e0826360/2020), having up to six mutations in the antigenic site B (Figure 2B). These antigenic site B mutations highly impacted the VE: the calculated efficacy using the P_epitope_ model was −23.6%.

The 2022/2023 flu season in Romania was characterized by the cocirculation of five different A/H3N2 viral clades. In the phylogenetic tree (Figure 2A), the sequences isolated in the 2022/2023 season are in red. The majority of sequences from this flu season belonged to clade 2b (91/164), with 53 sequences clustered in clade 2a.1b, 15 classified as clade 2a.3a.1, 3 belonging to clade 2a.1, and 2 others clustered in clade 2a.3b. Mutations detected in the main circulating clade 2b are presented in Figure 2B, and the impact on VE was estimated (VE = 21%). Forty-three of the sequences belonging to clade 2b presented one mutation in antigenic site A (I140K) and two in antigenic site B (S156H, N186D), reducing VE to 23.47%. All sequences belonging to clade 2a.1b had antigenic site C as the dominant epitope, with three mutations (Figure 2B) and a calculated VE of 18.5%. Three sequences from clade 2a.1b that circulated in Romania during the 2022/2023 flu season had an additional mutation in antigenic site A (I48M, K278R, or V297I), which reduced VE to 9%. Antigenic site C was also the dominant epitope for the sequences belonging to clade 2a.1.

A more diverse pattern of mutations in the antigenic sites was seen in the sequences belonging to clade 2a.3a.1. All the sequences had one mutation in antigenic site A, two in antigenic site B, two in antigenic site C, and one in antigenic site D (Figure 2B). Most of the sequences (8/15) had antigenic site B as the dominant epitope and a calculated VE of 23.47%. Six sequences had site A as the dominant epitope with two mutations (N122D and I140K), leading to a reduction in VE to 21%. The sequences belonging to clade 2a.3b had two mutations in antigenic site B, reducing VE to 23.47%. In addition to the mutations in the dominant epitopes, other mutations in antigenic epitopes were identified, which are presented in Figure 2B.

The diversity of influenza A(H3N2) observed in the 2023/2024 season (in magenta in Figure 2A) in Romania was low, characterized by the circulation of subclade 2a.3a.1 viruses (119/120). One of the sequenced samples (A/Romania/3420/2023) was unassigned by FluSurver software [25], as it was located at the root of clade 2a.3a.1 in the tree, lacking clade-defining mutations. The 2023/2024 sequences clustered with the sequence from the recommended 2024/2025 vaccine (A/Thailand/8/2022) and a few sequences that circulated during the 2022/2023 season (represented in red).

By comparing with the corresponding seasonal vaccine sequence (A/Darwin/9/2021), the sequences from this particular subclade presented a high number of mutations, affecting multiple antigenic sites.

Thus, three mutations were found in antigenic site A, four mutations in antigenic site B, four mutations in antigenic site C, five mutations in antigenic site D, and four mutations in antigenic site E (Figure 2B). Most of the sequences (65/119) presented antigenic site C as the dominant epitope, with three mutations (56/65; P_epitope_ = 0.1153), four mutations (8/65; P_epitope_ = 0.1538), or five mutations (1/65; P_epitope_ = 0.1923), reducing the VE to 18.5%, 9%, or 0%, respectively. Other sequences from this subclade (38/119) had antigenic site B as the dominant epitope, presenting two mutations (26/38; VE = 23.48%) or three mutations (12/38; VE = 11.71%). The other seventeen sequences had antigenic site A as the dominant epitope, with two mutations (4/17) or three mutations (13/17), reducing VE to 21% or 8%, respectively.

### 3.3. Influenza B Variability and Phylogenetic Relationship of Circulating Strains in Romania (2021–2024)

**Figure 3:**
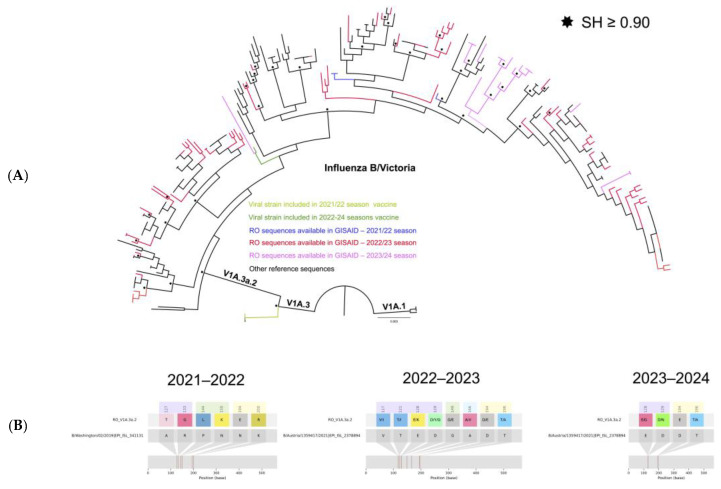
(**A**) Phylogenetic relatedness between influenza B virus sequences reported in Romania (2021–2024). Shimodaira Hasegawa (SH) support values higher than 0.9 are indicated at nodes with an asterisk *. Romanian (RO) sequences are depicted according to each season in blue (2021/2022), red (2022/2023), and magenta (2023/2024). (**B**) The HA amino acid variability of the circulating influenza B virus strains for each studied season. Antigenic epitopes are highlighted as follows: lavender (120-loop), green (150-loop), light blue (160-loop), and light yellow (190-helix).

A total of 101 influenza B virus strains circulating in Romania during 2021–2024 were sequenced and deposited in the GISAID database. Based on FluSurver analysis, all these sequences belonged to lineage Victoria, clade V1A.3a.2. The phylogenetic analysis (Figure 3) showed that all the Romanian sequences were part of the same cluster with the sequences from the recommended 2022/2023 and 2023/2024 vaccine strain (B/Austria/1359417/2021). Only four Romanian sequences were reported in the GISAID database during the 2021/2022 flu season and are depicted with a blue line in Figure 3. These variants possessed several mutations when compared with the 2021/2022 vaccine sequence (B/Washington/02/2019), as seen in Figure 3B. Based on the P_epitope_ model these mutations might reduce VE to 49%.

The mutational screening analysis of the sequences from the 2022/2023 season showed that 53 sequences out of 77 had one mutation (D194E) in the 190-helix, reducing the VE to 60%. Other mutations were also found for several sequences reported in Romania in this period of time in all four antigenic epitopes (Figure 3B). The estimated impact of these mutations on VE was rather low.

Low variability was also observed when analyzing influenza B sequences reported in Romania in the 2023/2024 season. Compared with the vaccine strain sequence (B/Austria/1359417/2021), the sequences collected in Romania in the 2023/2024 flu season showed low variability. Notably, 19 out of 20 sequences had one mutation in the 190-helix (D194E), and 16 presented one additional mutation in the antigenic site 120-loop (E128G for 9 sequences and D129N for 6 of them). Based on the P_epitope_ model, the impact of these mutations was rather low, reducing the VE to 59%.

## 4. Discussion

This study presents an extensive analysis of the HA gene of circulating influenza viruses among hospitalized patients in Romania; a validated mathematical model to calculate VE was used to assess the impact of influenza vaccination in this population.

Vaccination against influenza viruses remains the most effective way to reduce the disease burden, significantly reducing hospitalization and comorbidities, especially in risk groups such as young children and elderly people [26]. The currently available vaccines are mainly inducing neutralizing antibodies against the receptor binding domain (RBD) in the HA head domain. Influenza virus can escape the immune response by antigenic drift/shift. In addition to that, despite having a free vaccination program for risk groups (children, elders, healthcare workers, and people with chronic diseases), Romania fails to reach good vaccine coverage [12], and this might contribute to an increase in influenza virus circulation and the selection of escape mutations. This study showed that most of the viral variants reported in Romania during the last three cold seasons in this population with low vaccination coverage presented a number of mutations in HA antigenic sites, with an estimated negative impact on VE. Influenza A(H3N2) was the main subtype circulating during the 2021/2022 influenza season [15], similar to the previous season. These data are in accordance with previous data published in the European Region, showing a prevalence of more than 90% for influenza A virus strains, most of them subtype A(H3N2) [27,28,29]. In contrast with other European countries and the United States, where little diversity was reported during this season (clade 2a.2), this analysis indicated that influenza A/H3N2 sequences from Romania were distributed in multiple clades and subclades [27,30]. According to these results, the most prevalent circulating influenza A(H3N2) clade in Romania was 2a.1 (56%), followed by 2a.3b (17.5%). These strains were characterized by the presence of up to six mutations in the antigenic site B with a strong negative effect on VE, as estimated by the P_epitope_ model. The high variability conferred by the increased number of mutations identified in the A(H3N2) strains circulating in the 2021/2022 season could partly explain the increased number of infections with this particular subtype as compared to other influenza strains. Our data are in agreement with preliminary estimates published in the United States, which reported a 16% (95% CI = −16% to 39%) VE for influenza A(H3N2) in medically attended outpatients during that season [30]. Similar results were published in Italy for the 2021/2022 season, estimating the VE at −28.9% based on the P_epitope_ model, with antigenic site B being the dominant one [29]. The results obtained from a larger European cohort that included Romania (Development of Robust and Innovative Vaccine Effectiveness (DRIVE)) showed overall good vaccine effectiveness for the 2021–2022 season [14]. This observation should still be interpreted with caution due to the wide confidence intervals (CIs) reported in this study. In accordance with data published for the 2021/22 flu season in Europe and the US, which showed low circulation of influenza A(H1N1)pdm09 and B/Victoria [27,30], only few sequences of influenza A(H1N1)pdm09 and influenza B/Victoria lineage were reported in Romania for the same period; these sequences had several mutations in antigenic sites, which might have reduced VE according to our analysis with the P_epitope_ model.

During the 2022/2023 influenza season, both influenza A and B viruses were circulating; influenza A(H3N2) was the most prevalent according to both the European Centre for Disease Prevention and Control (ECDC) [27] and Centers for Disease Control and Prevention (CDC) [31]. In contrast, a higher prevalence of influenza A(H1N1) in Romania was observed in the present study. These differences might be explained by the distinct study designs and sampling strategies: the majority of the sequences for this season were collected in the NIID “Prof. Dr. Matei Balș”, where a great proportion of enrolled patients were hospitalized. Data from the GIHSN involving 73,121 patients hospitalized with respiratory illness from 22 countries showed similar results: infections with influenza A(H1N1) were more severe than A(H3N2) infections [16]. Most of the A(H1N1) viruses from the 2022/2023 season were part of clade 6B.1A.5a.2a and had antigenic site Sb as the dominant epitope, with two mutations, which might have reduced the VE to 33.17%. Our data are in accordance with interim 2022/2023 influenza vaccine effectiveness estimations from six studies that cover 16 countries, showing that vaccine effectiveness ranged from 28% to 46% for influenza A(H1N1). The majority of the A(H3N2) HA sequences collected during the 2022/2023 influenza season belonged to clade 2b, followed by clade 2a.1b and clade 2a.3a.1. Mutations in B-cell epitopes were found that made it distinct from the seasonal flu vaccine, suggesting that it might escape from neutralization by vaccine-induced antibodies, and the efficacy estimated with the P_epitope_ model underlined their impact. In agreement with this mathematical model, the estimations of vaccine effectiveness during this season ranged from 2% to 44%, being higher among children (62–70%) [16]. The highest vaccine effectiveness was reported for influenza B viruses [16], due to the lowest mutation rates detected in antigenic epitopes.

The 2023/2024 influenza season in Europe was characterized by a high prevalence of influenza A virus, especially A(H1N1), and low circulation of influenza B viruses [32]. This was also valid for Romania, where most of the samples analyzed were subtype A(H1N1). The A/H1N1 sequences clustered in two clades, 6B.1A.5a.2a and 6B.1A.5a.2a.1 (2/21 clustered with the vaccine strain). Two mutations in the antigenic site Ca2 of clade 5a.2a strains might explain the VE reduction. The A(H3N2) sequences from the 2023/2024 influenza season clustered in clade 2a.3a.1 and showed high variability in the antigenic sites compared to the sequence of the vaccine strain. Several sets of mutations were identified in these sequences as compared with the strain included in the seasonal vaccine, with a reduction in the VE as estimated by the P_epitope_ model. Influenza B viruses circulating during this season presented low variability in antigenic sites, with a small impact on VE. A multicenter study focused on 2023/2024 influenza A vaccine effectiveness, including both primary care and hospital sites, and showed vaccine effectiveness for hospital settings of 44% (95% CI: 30–50%) for H1N1 and 14% (95% CI: −32–43%) for H3N2, similar to our report [33]. Moreover, most of the strains reported here were collected from one hospital site, with more than half circulating in children. When analyzing the factors associated with more severe disease evolution (e.g., acute respiratory failure) [34], we observed that infection with influenza B viruses caused less acute respiratory failure as compared to influenza A virus, underlining the need for a sharper focus on influenza A in order to limit the disease burden.

The low vaccine coverage reported by the Romanian Public Health Institute [12] might have contributed to this high variability of clades circulating in Romania during these previous seasons. In the case of influenza A viruses, some of the circulating viral strains were targeted by previous vaccine formulation, but in an unvaccinated population, these clades continue to spread during subsequent seasons.

Currently, there are several strategies that are believed to improve the influenza vaccine toward a more efficient, universal version. The COVID-19 pandemic has led to the development of new vaccine formulations that might be fructified also for other pathogens in order to improve efficiency. New approaches to improve current influenza vaccine formulation are considered, from the use of adjuvants to targeting the stem region of hemagglutinin [35,36,37,38]. Moreover, the immunogenicity of a new formulation (hexavalent influenza vaccine) is currently evaluated in clinical trials [39]. However, prior exposure to influenza viruses (either after infection or by vaccination) was shown to impact vaccine effectiveness; the antigenic distance between the circulating virus and the vaccine component seems to be an important contributing factor [40].

## 5. Conclusions

Our data show the high variability of influenza strains circulating during the last three seasons, with a mutational pattern that might partly explain the viral escape from antibodies produced by vaccination. Influenza vaccine is able to generate antibodies that protect mainly against the inducing strain; it is difficult to achieve good infection control by vaccination in the context of highly diverse circulating strains. Our data underline the need for public health programs to increase vaccine uptake and improve vaccine formulation in order to limit viral transmission.

## Data Availability

The datasets generated and analyzed during the current study are available from the corresponding author upon reasonable request.
